# Naltrexone Reverses Ethanol Preference and Protein Kinase C Activation in *Drosophila melanogaster*

**DOI:** 10.3389/fphys.2018.00175

**Published:** 2018-03-14

**Authors:** Rajeswari Koyyada, Nilesh Latchooman, Julius Jonaitis, Samir S. Ayoub, Olivia Corcoran, Stefano O. Casalotti

**Affiliations:** Medicines Research Group, School of Health, Sport and Bioscience, University of East London, London, United Kingdom

**Keywords:** ethanol, CAFE assay, PKC, opiate antagonist, *Drosophila melanogaster*, naltrexone

## Abstract

Alcohol use disorder (AUD) is a major health, social and economic problem for which there are few effective treatments. The opiate antagonist naltrexone is currently prescribed clinically with mixed success. We have used naltrexone in an established behavioral assay (CAFE) in *Drosophila melanogaster* that measures the flies' preference for ethanol-containing food. We have confirmed that *Drosophila* exposed to ethanol develop a preference toward this drug and we demonstrate that naltrexone, in a dose dependant manner, reverses the ethanol-induced ethanol preference. This effect is not permanent, as preference for alcohol returns after discontinuing naltrexone. Additionally, naltrexone reduced the alcohol-induced increase in protein kinase C activity. These findings are of interest because they confirm that *Drosophila* is a useful model for studying human responses to addictive drugs. Additionally because of the lack of a closely conserved opiate system in insects, our results could either indicate that a functionally related system does exist in insects or that in insects, and potentially also in mammals, naltrexone binds to alternative sites. Identifying such sites could lead to improved treatment strategies for AUD.

## Introduction

Alcohol abuse and alcohol use disorder (AUD, commonly referred to as alcohol addiction) are global health problems with major social, mental health, and economic consequences (Gilmore et al., 2016). AUD is a complex disease affected by both genetic and environmental factors (Flatscher-Bader and Wilce, 2009). The molecular mechanisms resulting from alcohol consumption and leading to alcohol use disorder are still not completely understood. Clinically, AUD is currently treated with mixed success using both psychological and drug therapies. With respect to the latter, acamprosate (Kufahl et al., 2014), naltrexone (Hendershot et al., 2016) and more recently nalmefene (Soyka, 2016) have been the most widely used drugs for treating AUD. However, clinical studies have yet to conclusively demonstrate the general effectiveness of these drugs (Arias and Sewell, 2012). Naltrexone is an opiate antagonist believed to exert its action on alcohol craving and relapse by blocking the μ opiate receptors which are involved in the molecular mechanisms of addiction (Gilpin and Koob, 2008). Although the mechanisms of ethanol induced behavioral changes are not well understood, it is known that ethanol alters the function of a number of neurotransmitters receptors (Liang and Olsen, 2014) and affects signal transduction including an increase in Protein Kinase C activity (Wilkie et al., 2007), which in turn also affects neurotransmitter receptors (Kumar et al., 2006).

Opiate peptides and receptors have been implicated in addiction mechanisms in response to many psychoactive substances including alcohol (Koob and Volkow, 2016). However, the potential of using opiate receptors as a therapeutic target for AUD remains controversial and indeed the use of naltrexone and nalmefene in the clinic has arisen from empirical observations rather than an understanding of their mechanism of action.

A variety of rodent models have been developed to try dissecting the molecular components of addictive behaviors (Crabbe, 2014). The fruit fly *Drosophila melanogaster* has proven to offer several advantages which include displaying simple alcohol-induced behaviors such as motor impairment and sedation, and the availability of a wide range of mutants for both reverse and forward genetics (Devineni and Heberlein, 2013; Park et al., 2017). *Drosophila* have an intrinsic capacity of sensing alcohol and indeed, identifying alcohol sources in rotting fruit, is part of the female's egg-laying strategy when deciding where to position the eggs for the maximal benefit to the larvae (Yang et al., 2008). Several studies have shown that when flies are repeatedly exposed to ethanol levels of up to 10–15% they develop a behavior that suggests that the flies have had a rewarding experience and that they seek more ethanol (Devineni and Heberlein, 2013; Peru Y Colón de Portugal et al., 2014). The capillary feeder assay (CAFE) is a convenient method for assessing the flies preference for alcohol (Ja et al., 2007) and was used here to determine whether naltrexone could alter the observed development of preference toward alcohol-containing food.

The choice of *Drosophila* for this study may seem controversial due to the lack of evidence for mammalian-like opiate systems in *Drosophila* or indeed in insects and other invertebrates. Recently however, behavioral effects of morphine have been reported in ants (Entler et al., 2016), crayfish (Huber et al., 2011), and *C. elegans* (Cheong et al., 2015). Additionally, two G-protein coupled receptors with structural homology to mammalian opioid/somatostatin receptors, but activated by allatostatin-like peptides, have been described in *Drosophila* (Lenz et al., 2000; Kreienkamp et al., 2002). The existence of these opiate-like systems which may have different activators or effectors, but result in similar behaviors, is in itself an important area of investigation because it may elucidate novel mechanisms in mammalian systems.

We show here that naltrexone reduces the preference for consumption of alcohol-containing food in flies previously exposed to alcohol and in the same flies it reduces the alcohol-induced increase of Protein Kinase C (PKC) activity. This study thus reinforces the need to further investigate novel targets or mechanism of action for opiate antagonists in treating AUD.

## Materials and methods

### Fly maintenance

Wild type Drosophila Canton S were obtained from Bloomington Centre (Stock 64349) and maintained at 24°C, 70% humidity 12 h light/dark cycle on ready made mixed dried food (Batch no: B8A03876 obtained from Phillip Harris). For all experiments 1–3 day old male flies were used.

### CAFE assay

The previously described CAFE method was adopted (Ja et al., 2007). The CAFE apparatus consisted of 9 × 1.5 cm (height × diameter) tubes where the fly chamber was limited by inserting a cotton plug (flugs, Dutscher cat 789036) to create two chambers within the tube. To provide humidity, water (2 ml) was added to the lower chamber through a small hole created with a hot needle and plugged with plasticine. The top chamber was 5 cm high and hosted the flies. All incubations were carried out in the incubator at 24°C, 70% humidity. Four 5μ*l* capillary tubes (cat: CAP-TF-5 Jaytec Glass Ltd UK) were inserted in the top flug via cut-off pipette tips. Liquid food (5% Sucrose w/v, 5% w/v yeast extract) with or without 15% ethanol or naltrexone was loaded into the capillary tubes. Eight 1–3 day old male flies were anesthetized with CO_2_ and placed in the chamber. Occasionally during the whole treatment some flies died, tubes with less than six flies were discarded. Flies were fed via capillaries for 2 days with liquid food with or without ethanol (pre-treatment). The duration of pre-treatment (48 h) and the concentration of ethanol (15%) were chosen after initial optimization for maximum preference response and are consistent with other reports (Ja et al., 2007; Devineni and Heberlein, 2013). Capillaries were reloaded with food or food plus naltrexone for 24 h. Capillaries were removed for 24 h. During this starvation period humidity was maintained by the presence of water in the lower chamber. Starvation increases consumption during the assay and reduces variability between groups. Four capillaries reintroduced where two capillaries contained food and the other two contained food plus 15% ethanol. The amount of food consumed was measured in the same batch of flies after 2 and 24 h by placing each capillary tube under a dissecting microscope aligned to a ruler with millimeter divisions. A tube containing no flies was used as control for liquid evaporation and the values were subtracted from the experimental tubes (corrected values). The preference index was calculated as the ((corrected ethanol consumption) − (corrected food consumption))/(corrected total consumption). Variations of the above protocol are described in the text.

### Protein kinase C assay

Protein Kinase C (PKC) activity was measured using the kit from Abcam UK (cat 789036). This is an ELISA-based system where a peptide with the specific substrate sequence for the PKC protein family is immobilized on the walls of the microtiter plate wells. Samples putatively containing PKC are incubated in the wells. Antibodies specifically recognizing the phosphorylated form of the immobilized peptides are added and detected by enzyme-linked secondary antibodies. Flies were fed via capillary tubes with either just food (prepared as above), or food with 15% ethanol for 48 h. Flies were then either exposed to food or food and 0.1% naltrexone for 24 h and then sacrificed by snap freezing in liquid nitrogen. Fly heads were separated by vortexing and homogenized in lysis buffer [20 mM MOPS, 50 mM β-glycerolphosphate, 50 mM sodium fluoride, 1 mM sodium vanadate, 5 mM EGTA, 2 mM EDTA, 1% NP40, 1 mM dithiothreitol (DTT), 1 mM benzamidine, 1 mM phenylmethanesulphonylfluoride (PMSF)] and either stored at −20°C or used immediately according to the manufacturer's instructions. Absorbance of each well was measured in a microtiter plate scanner. The protein content of the samples were estimated by a Bradford assay using bovine serum albumin as a standard. The specific activity of protein kinase C was calculated as absorbance value of the ELISA assay divided by absorbance value of the protein assay.

### Statistical calculations

**Figure 1:**
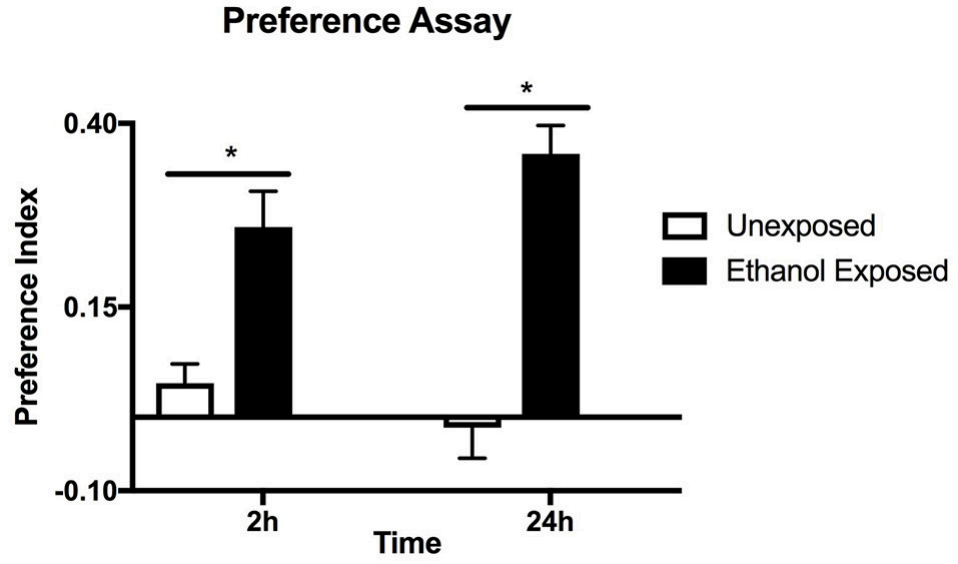
Preference assay for naive or ethanol exposed flies. Preference indices were measured at 2 and 24 h in the same batch of flies. Columns represent three independent experiments, each consisting of three assay tubes containing 6–8 flies each. *n* = 9. Error bars are SEM. Statistical significance was measured by two-way ANOVA with Bonferroni multiple comparisons test. The effect of alcohol treatment was highly significant *p* < 0.0001 (horizontal line with^*^). The effect of time was not significant *p* = 0.635.

**Figure 2:**
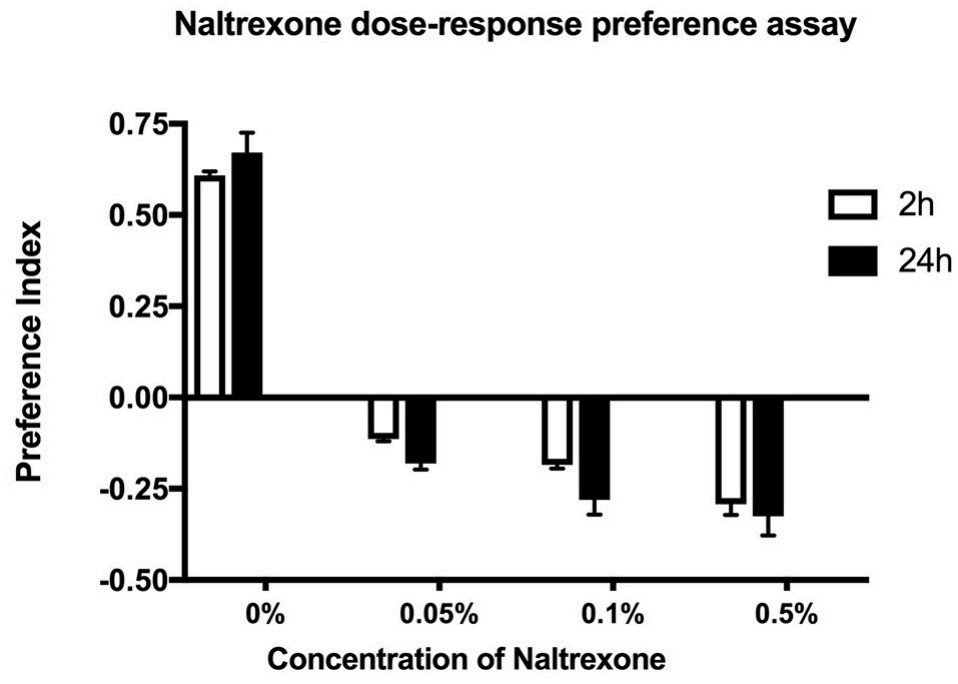
Concentration dependent effect. Preference assay for ethanol exposed flies fed with naltrexone (0–0.5% w/v) for 24 h before being starved for 24 h and tested for ethanol preference in the CAFE assay. Preference indices measured at 2 and 24 h. Columns represent three independent experiments, each consisting of three assay tubes containing 6–8 flies each. *n* = 9. Error bars are SEM. Statistical significance was measured by two-way ANOVA with Bonferroni multiple comparisons test. The effect of naltrexone treatment was overall highly significant *p* < 0.0001 but there was no statistical difference between consecutive naltrexone concentrations tested. The effect of time was not significant *p* = 0.175.

**Figure 3:**
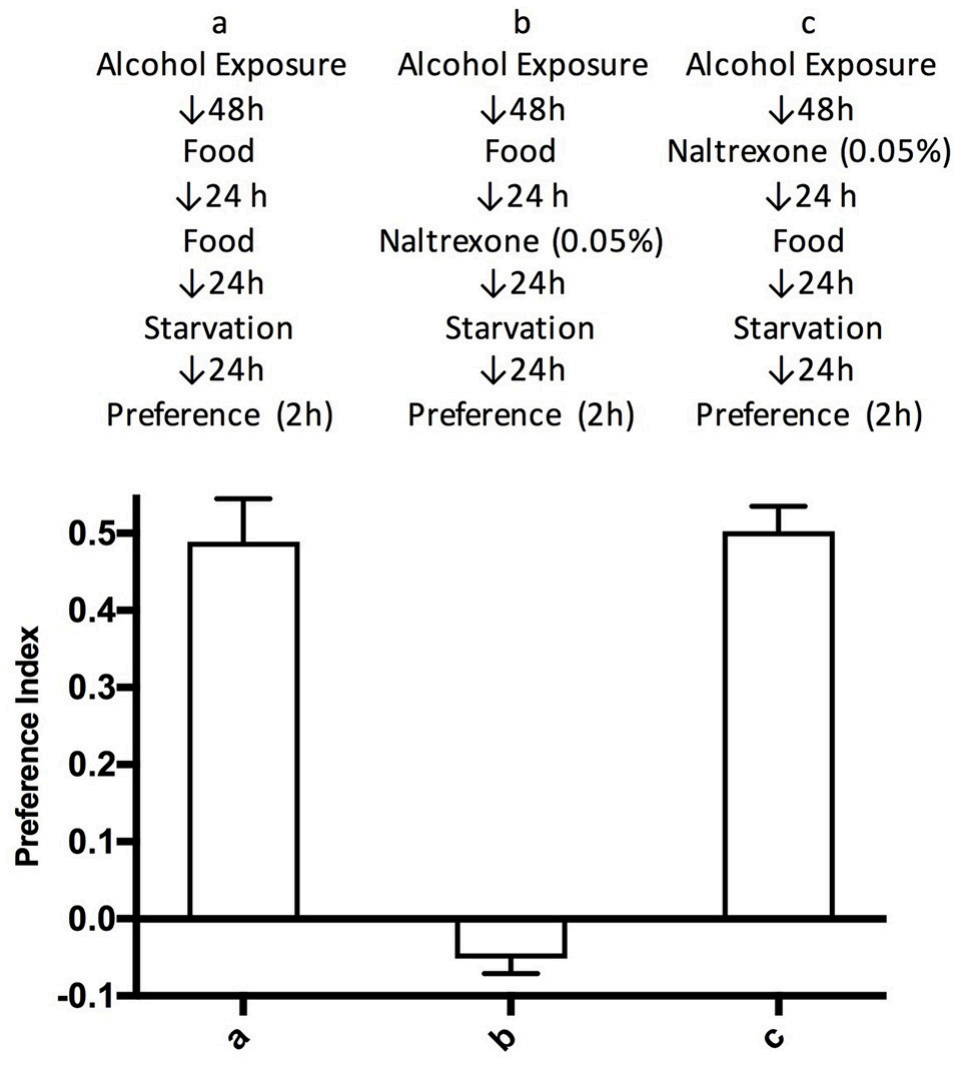
Time duration of naltrexone effect. Flies were treated according to schemes a–c described in the top part of the figure. Preference indices were measured after 2 h. Each bar represents three experiments with triplicate assays containing 9 flies each, *n* = 9. Error bars are SEM. One-way ANOVA with Bonferroni multiple comparison analysis showed a significant difference between group a and b, and b and c, *p* < 0.001, but not between a and c with *p* = 0.506.

**Figure 4:**
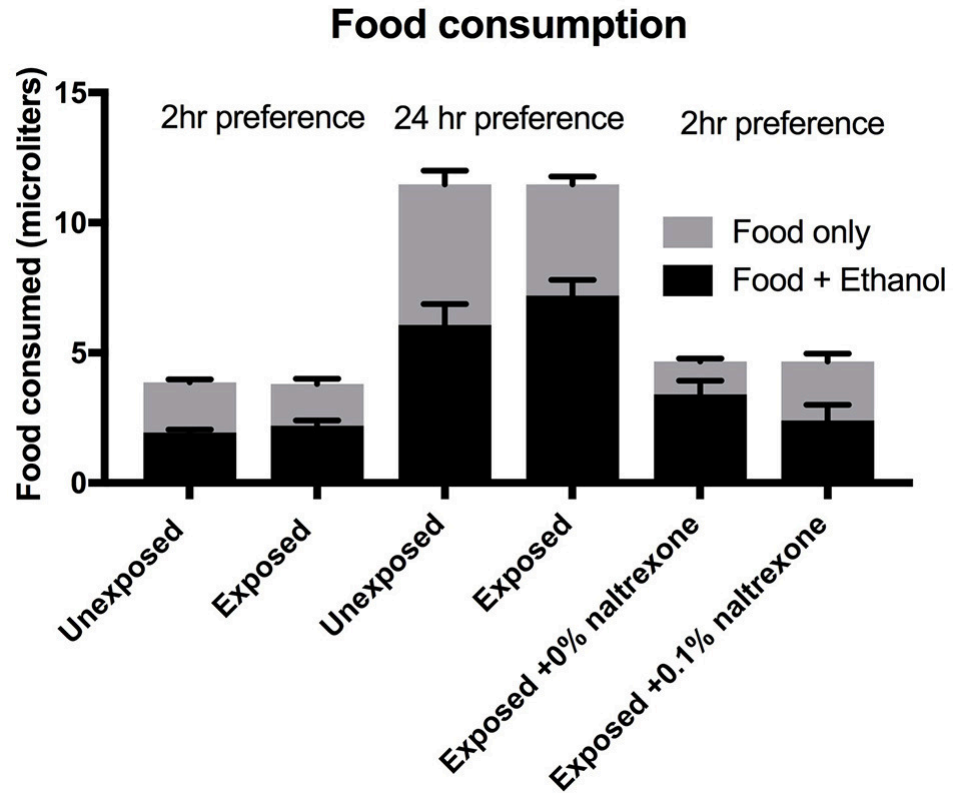
Food consumption. Flies were either exposed or unexposed to 15% ethanol for 48 h or exposed to ethanol for 48 h followed by treatment with 0 or 0.1% naltrexone. The consumption was measured over a period of 2 or 24 h as indicated. Each column represents triplicate assays containing 6 flies each, *n* = 3. Error bars are SEM. One-way ANOVA with Kruskall-Wallis multiple comparison analysis showed no significant difference (*p* > 0.9) between any of the groups where consumption was measured for the same length of time. The Preference indices for the data shown for this figure for the 6 columns left to right are 0.0; 0.16; 0.02; 0.26; 0.48; 0.03.

**Figure 5:**
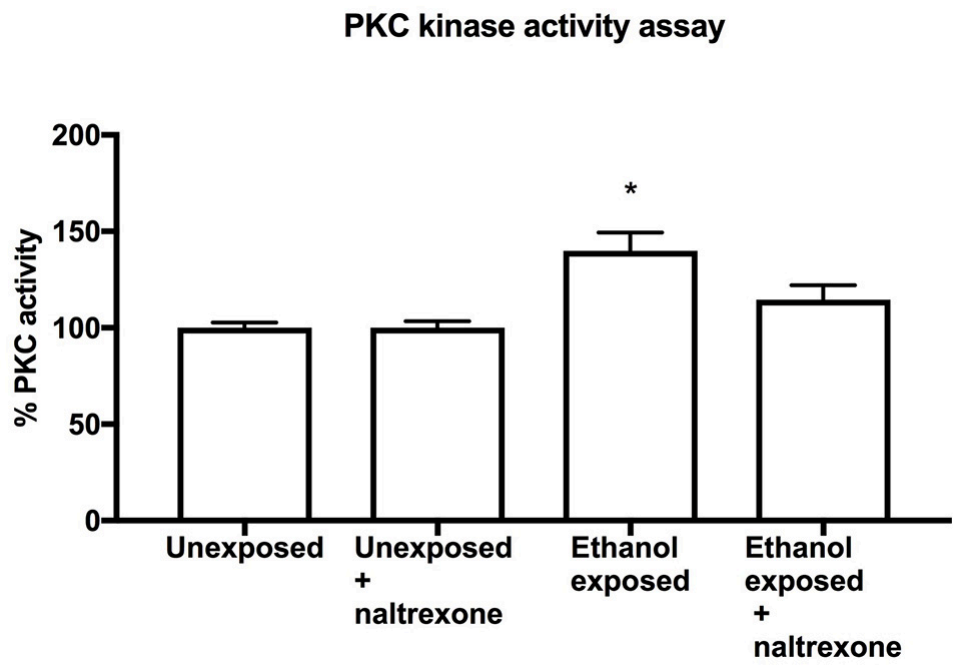
PKC Kinase activity assay. PKC assay of homogenates from heads of flies exposed to food for 72 h (Unexposed), exposed to food for 48 h and treated with naltrexone for 24 h (Unexposed + naltrexone) exposed to ethanol for 48 h and food for 24 h (Ethanol exposed) or exposed to ethanol 48 h followed by naltrexone for 24 h (Ethanol exposed + naltrexone). Each bar represent two independent experiments each consisting of triplicate assays containing 15 fly heads each. *n* = 6. Error bars are SEM. Data was analyzed by non-parametric one way ANOVA Kruskal-Wallis with *post-hoc* comparison to Unexposed flies. The only statistical difference was between Unexposed and Ethanol exposed ^*^p = 0.037.

Data was analyzed with the statistical package Graph Pad. Data were first analyzed for normal distribution by Shapiro-Wilk normality test. If it passed the normality test (alpha = 0.05) parametric tests were used (Figures 1–**3**) alternatively non-parametric tests were used (**Figures 4**, **5**) A preference index calculated from one tube containing 6–8 flies was considered as *n* = 1. Results were considered statistically significant if *p* < 0.05.

## Results

### Alcohol preference is induced by ethanol pre-exposure and inhibited by naltrexone

*Drosophila* were housed in the CAFE apparatus for 2 days and either fed liquid food or, in separate tubes, liquid food with 15% ethanol. After a 24 h starvation period flies were offered a choice of food with and without ethanol and the consumption from the capillary tubes was measured at 2 and 24 h (Figure 1). Flies with previous exposure to ethanol showed preference for ethanol-containing food, unlike the naive flies. Similar levels of preference were observed whether the first 2 or 24 h of food consumption were measured suggesting that the effect is due to the pre-exposure to ethanol rather than familiarity with the apparatus during the assay. The assay is thus measuring an established rather than a developing behavior.

To test the effect of naltrexone on alcohol preference, ethanol pre-exposed flies were fed food containing 0.05–0.5% naltrexone for 24 h, then starved for 24 h before testing for alcohol preference in the CAFE assay (Figure 2). The naltrexone dose range was chosen to include approximate equivalent values of the mg/kg bodyweight amounts used in mammalian systems (Critcher et al., 1983). The results in Figure 2 indicate that naltrexone had an overall significant (*p* < 0.0001) effect in reversing ethanol preference however there was no significant difference between adjacent doses tested. This result suggests that naltrexone acts on a specific target to induce its effect. High doses of naltrexone appeared to cause an avoidance of ethanol (negative preference values shown in Figure 2) however, naive flies exposed to 0.1% naltrexone did not show negative preference (data not shown), thus the effect of naltrexone appears to be related to the response to ethanol.

### The effect of naltrexone on alcohol preference is not permanent

To test whether naltrexone permanently reverses alcohol preference in *Drosophila* we introduced an additional step in the treatment of the flies whereby after the naltrexone treatment (0.05%), flies were fed normal food for 24 h before being starved and tested in the CAFE assay. This was carried out to allow naltrexone to be fully metabolized and thus presumably being absent during the CAFE assay. Flies treated in this manner showed preference for alcohol equal to those never exposed to naltrexone, while as previously shown in Figure 2, in the flies tested in the CAFE assay within 24 h of the end of the naltrexone treatment, the preference for ethanol was no longer detectable (Figure 3).

### Total food consumption is not affected by ethanol or naltrexone

To emphasize the concept that ethanol alters a decision making process (preference) rather than an instinctive physiological behavior (food consumption) we present the data for total food consumption, i.e., the sum of “food only” and “food + ethanol” consumed by the flies in each vial. The data shown in Figure 4 is derived from sets of triplicate assays carried out on the same batch of flies for each experiment. No significant difference (*p* > 0.9) can be observed between the total food consumptions when comparing flies exposed or not exposed to ethanol(15%) whether the preference assay is carried out for 2 or 24 h or when comparing flies exposed to ethanol (15%) alone with flies exposed to ethanol(15%) and naltrexone (Figure 4). We did observe some variation between batches of flies: for example the total food consumption of the flies labeled as “exposed to ethanol and 0% naltrexone” is slightly higher (but not significantly, *p* = 0.99) than flies labeled as “ethanol exposed” which is the effectively the same treatment. These small variations in total food consumption occur between different batches of flies and may be due to factors such as age distribution (all flies are between 0 and 5 days old at the start of the experiment) room temperature, small differences in the time of the day the experiment is carried out. However, despite these small insignificant variations in total food consumption (see Supplementary Table 1), we consistently observe significant changes in the preference index induced by ethanol and suppressed by naltrexone as shown in Figures 1–3.

### Naltrexone affects ethanol-induced PKC activity

In order to broaden the investigation of the behavioral effect of naltrexonon alcohol induced events, we chose to biochemically investigate the known phenomenon of the increase of level of PKC following ethanol stimulation. Using an ELISA assay to measure PKC activity in fly head extracts, we have confirmed that like in mammals, ethanol consumption (food with 15% ethanol for 48 h followed by food only for 24 h) induced a statistically significant increase (*p*= 0.037) in PKC activity (Figure 4). However, flies exposed to naltrexone (food with 15% ethanol for 48 h followed by food with 0.1% naltrexone for 24 h) showed no statistically significant increase in PKC, indicating that naltrexone affected the ethanol-induced increase in PKC activity. Flies exposed to naltrexone alone, in the absence of any alcohol treatment, showed no change in basal PKC activity (Figure 5).

## Discussion

In this work we have used the CAFE assay (Ja et al., 2007) to study long term *Drosophila* response to alcohol consumption. We have confirmed that preference for ethanol-containing food is induced by previous exposure to ethanol as opposed to being induced by preference in taste or immediate reward, because naive flies that were exposed to the ethanol-containing food for 24 h did not show significant preference (Figure 1). The novel aspect of our work is that we have provided evidence that the opioid antagonist naltrexone can neutralize ethanol preference in flies previously exposed to alcohol. The overall effect of naltrexone was dose dependant and at higher doses naltrexone caused a negative preference (repulsion) for ethanol-containing food (Figure 2). It is not possible to conclude from these experiments whether the reduction of preference and the induction of aversion are part of the same phenomena or are two separate processes requiring different concentrations of naltrexone.

The effect of naltrexone appeared to be short lived: ethanol pre-exposed flies that were allowed to recover a total of 48 h (24 h food + 24 h starvation) after naltrexone treatment before being tested in the CAFE assay, showed the same level of ethanol preference as flies that had not been exposed to naltrexone (Figure 3). The fact that the ethanol-induced alcohol preference is longer lived than the effect of naltrexone would suggest that while alcohol has a chronic effect that persists beyond the time in which alcohol is still present in the system, naltrexone has a more acute effect. This would suggest that either naltrexone activates a system that counteracts the alcohol-induced effect or that naltrexone antagonizes an opiate-like system that is an integral part of the development of the alcohol-induced alcohol preference. From the behavioral experiments presented here it is not yet possible to determine the exact mechanism of action of naltrexone in *Drosophila*. To exclude possible confounding factors we have observed that ethanol treatment with or without naltrexone did not affect the total amount of food consumed when the flies were given the choice of food with or without ethanol; indeed the total consumption of any food at any stage of the experiment showed no significant variations (Figure 4). Additionally, administration of naltrexone prior to the initial 48 h ethanol exposure did not affect the induction of ethanol preference (data not shown). It thus appears that naltrexone affects preferentially the behavioral seeking of ethanol in ethanol-exposed flies. The dopaminergic system is known to be implicated in addictive mechanisms in *Drosophila* (Azanchi et al., 2013; Aranda et al., 2017) and in the mammalian nervous system this is influenced by the opiate system (Koob and Volkow, 2016), further work is required to investigate this relationship in *Drosophila*.

The underlying theory of addiction behavior is that psychoactive substances cause long term changes at the cellular and molecular level which then result in behavioral changes (Nestler, 2014). To investigate whether naltrexone altered any of the known ethanol-induced biochemical changes we chose to investigate its impact on PKC activation. In mammals, chronic ethanol exposure causes an increase in PKC activity (Wilkie et al., 2007) while in *Drosophila* inactivation of PKC genes cause a desensitization to ethanol (Chen et al., 2010). Our data indicates that PKC phosphorylation is elevated in flies exposed to ethanol as compared to naive flies. This result, which to our knowledge is the first direct measurement of ethanol-induced PKC increase in *Drosophila*, further justifies the use of *Drosophila* as a model for the study of mammalian addiction mechanisms. Moreover, we demonstrate here that naltrexone affected the ethanol-induced increase of PKC to the extent that in naltrexone-treated ethanol-exposed flies PKC activity was no longer significantly different from unexposed flies (Figure 5). It should be noted that the specificity for PKC in this assay is based on the sequence of the peptide immobilized on the ELISA plates, it is possible that other kinases may have contributed to the phosphorylation process. The results shown in this study do not provide details of the mechanism of action for naltrexone with respect to PKC activity, but confirm the ability of naltrexone to alter alcohol-induced phenomena. Previous work in mammalian systems on the effect of naltrexone on PKC have reported an increase in PKC expression (Yu et al., 2011) and an antagonistic effect on ethanol induced increase of PKC activity (Oh et al., 2006). While further elucidating the role of PKC in addiction processes would be of interest, our aim for this study was to demonstrate that naltrexone reduces both an alcohol-induced behavior (ethanol-induced alcohol preference) and an alcohol-induced biochemical process (ethanol-induced increase in PKC activity). Taken together these findings justify further work to investigate the mechanism of action of naltrexone in *Drosophila* and in mammalian systems. Indeed, it would also be of interest to understand how the putative naltrexone response system interacts with the dopaminergic system which is known to be involved in addiction behaviors and other related functions such as memory (Kaun and Rothenfluh, 2017) and circadian rhythms (De Nobrega and Lyons, 2016).

Understanding the mechanism of action of naltrexone in *Drosophila* is complicated by the fact that unlike other mammalian neurotransmitter receptors, the opioid receptors are not highly conserved in *Drosophila*. Two opioid/somatostatin-like receptors Drostar-1 and -2 and their endogenous allatostatin-like peptides have been identified in *Drosophila* (Lenz et al., 2000; Kreienkamp et al., 2002), however further work would be required to investigate whether naltrexone interacts with drostar receptors which do not respond to mammalian opiate peptides (Kreienkamp et al., 2002). The implication of this work is that either naltrexone binds in *Drosophila* to an as yet unidentified receptor which is functionally but not structurally related to mammalian opiate receptors or that naltrexone operates through another target and mechanism in *Drosophila*. In the latter case it would be of interest to identify such a *Drosophila* target as there may be an homologous mammalian target that could help elucidate the mechanism of action of naltrexone and possibly be a target for improved treatment of AUD.

## Author contributions

RK obtained most of the data presented, NL contributed to the initial idea of using naltrexone, JJ and NL carried out initial experiments to develop the techniques, SA and OC contributed ideas to the project, analyzed the results and edited the manuscript, SC lead the project and wrote the manuscript. All authors reviewed the manuscript.

### Conflict of interest statement

The authors declare that the research was conducted in the absence of any commercial or financial relationships that could be construed as a potential conflict of interest.
