# Supplementary material for: Naltrexone Reverses Ethanol Preference and Protein Kinase C Activation in Drosophila melanogaster
Source: Front Physiol. 2018 Mar 14;9:175. doi: 10.3389/fphys.2018.00175 (PMC5861182; doi:10.3389/fphys.2018.00175)
Supplement: Supplementary file 1 [file DataSheet1.pdf]

Treatment	repeat	Food + Alcohol			food			total			PI 1	PI 2	PI 3	Average PI experiment
Unexposed 2hr preference fig1	1	2	1.8	2	1.8	2	2	3.8	3.8	4.0	0.053	-0.053	0.000	0.000
Unexposed 2hr preference fig1	2	1.9	2.1	2.3	1.9	1.8	2	3.8	3.9	4.3	0.000	0.077	0.070	0.049
Unexposed 2hr preference fig1	3	2.4	2.2	2.2	1.8	1.9	2	4.2	4.1	4.2	0.143	0.073	0.048	0.088
Exposed 2hr preference fig1	1	2	2.2	2.4	1.4	1.8	1.6	3.4	4.0	4.0	0.176	0.100	0.200	0.159
Exposed 2hr preference fig1	2	2.4	2.7	2.3	1.3	1.1	1.3	3.7	3.8	3.6	0.297	0.421	0.278	0.332
Exposed 2hr preference fig1	3	2.3	2.3	2.2	1.2	1.3	1.3	3.5	3.6	3.5	0.314	0.278	0.257	0.283
Unexposed 24 hr preference fig1	1	5.6	7	5.6	4.8	5.8	5.6	10.4	12.8	11.2	0.077	0.094	0.000	0.057
Unexposed 24 hr preference fig1	2	5.6	5.4	5.4	5.8	5.4	5.4	11.4	10.8	10.8	-0.018	0.000	0.000	-0.006
Unexposed 24 hr preference fig1	3	4.9	5	5.1	5.8	6.3	5.9	10.7	11.3	11.0	-0.084	-0.115	-0.073	-0.091
Exposed 24 hr preference fig1	1	7.2	7.8	6.6	4.2	4	4.6	11.4	11.8	11.2	0.263	0.322	0.179	0.255
Exposed 24 hr preference fig1	2	7.7	7.9	7.8	3.3	3.7	3.6	11.0	11.6	11.4	0.400	0.362	0.368	0.377
Exposed 24 hr preference fig1	3	7.9	8.1	8.4	3.1	3.5	3.6	11.0	11.6	12.0	0.436	0.397	0.400	0.411
Exposed +0% naltrexone 2hr preference fig2	1	4.5	4.3	3.9	0.9	1.1	1.2	5.4	5.4	5.1	0.667	0.593	0.529	0.596
Exposed +0% naltrexone 2hr preference fig2	2	4.3	4.3	3.9	1.1	1	0.9	5.4	5.3	4.8	0.593	0.623	0.625	0.613
Exposed +0% naltrexone 2hr preference fig2	3	4.2	4.3	4.1	1	1	0.9	5.2	5.3	5.0	0.615	0.623	0.640	0.626
Exposed +0.05% naltrexone 2hr preference fig2	1	2.1	2.4	2.4	3	2.9	2.8	5.1	5.3	5.2	-0.176	-0.094	-0.077	-0.116
Exposed +0.05% naltrexone 2hr preference fig2	2	2.1	2.2	2.3	2.8	3	2.7	4.9	5.2	5.0	-0.143	-0.154	-0.080	-0.126
Exposed +0.05% naltrexone 2hr preference fig2	3	1.9	2.1	2.3	2.6	2.7	2.5	4.5	4.8	4.8	-0.156	-0.125	-0.042	-0.107
Exposed +0.1% naltrexone 2hr preference fig2	1	1.7	2.5	2.1	3.2	2.9	2.6	4.9	5.4	4.7	-0.306	-0.074	-0.106	-0.162
Exposed +0.1% naltrexone 2hr preference fig2	2	2.2	1.9	2.5	3.5	3.2	3.1	5.7	5.1	5.6	-0.228	-0.255	-0.107	-0.197
Exposed +0.1% naltrexone 2hr preference fig2	3	2.4	1.7	2.1	2.6	2.8	3.6	5.0	4.5	5.7	-0.040	-0.244	-0.263	-0.183
Exposed +0.5% naltrexone 2hr preference fig2	1	1.9	1.3	1.8	3.8	3.5	3.1	5.7	4.8	4.9	-0.333	-0.458	-0.265	-0.352
Exposed +0.5% naltrexone 2hr preference fig2	2	2.1	1.9	1.7	2.7	3.6	3.3	4.8	5.5	5.0	-0.125	-0.309	-0.320	-0.251
Exposed +0.5% naltrexone 2hr preference fig2	3	2.5	1.7	1.6	3.3	3.2	3.5	5.8	4.9	5.1	-0.138	-0.306	-0.373	-0.272
Exposed +0% naltrexone 24hr preference fig2	1	11.1	9.3	9.7	1.1	1.4	1.5	12.2	10.7	11.2	0.820	0.738	0.732	0.763
Exposed +0% naltrexone 24hr preference fig2	2	10.2	9.7	10.2	1.9	1.7	2	12.1	11.4	12.2	0.686	0.702	0.672	0.687
Exposed +0% naltrexone 24hr preference fig2	3	9.8	10.3	10.1	4.1	2.3	1.9	13.9	12.6	12.0	0.410	0.635	0.683	0.576
Exposed +0.05% naltrexone 24hr preference fig2	1	6.1	5.5	4.7	7.7	8.5	8.1	13.8	14.0	12.8	-0.116	-0.214	-0.266	-0.199
Exposed +0.05% naltrexone 24hr preference fig2	2	6.3	5.1	4.8	7.9	7.8	8.7	14.2	12.9	13.5	-0.113	-0.209	-0.289	-0.204
Exposed +0.05% naltrexone 24hr preference fig2	3	6.5	5.7	5.2	8.4	6.9	8	14.9	12.6	13.2	-0.128	-0.095	-0.212	-0.145
Exposed +0.1% naltrexone 24hr preference fig2	1	5.3	5.9	3.5	8.2	7.4	9	13.5	13.3	12.5	-0.215	-0.113	-0.440	-0.256
Exposed +0.1% naltrexone 24hr preference fig2	2	6.1	6.2	3.8	7.9	8.4	9.1	14.0	14.6	12.9	-0.129	-0.151	-0.411	-0.230
Exposed +0.1% naltrexone 24hr preference fig2	3	3.3	5.7	3.5	10.2	7.8	9	13.5	13.5	12.5	-0.511	-0.156	-0.440	-0.369
Exposed +0.5% naltrexone 24hr preference fig2	1	5.3	5.7	4.7	8.4	9.2	8.1	13.7	14.9	12.8	-0.226	-0.235	-0.266	-0.242
Exposed +0.5% naltrexone 24hr preference fig2	2	4.2	3.3	4.5	9.8	11.2	9	14.0	14.5	13.5	-0.400	-0.545	-0.333	-0.426
Exposed +0.5% naltrexone 24hr preference fig2	3	3.5	4.8	6	9.1	8.8	9.2	12.6	13.6	15.2	-0.444	-0.294	-0.211	-0.316
Exposed +0 % naltrexone (fig 3 a)	1	4.3	3.7	2.9	1.7	1.1	0.9	6.0	4.8	3.8	0.433	0.542	0.526	0.500
Exposed +0 % naltrexone (fig 3 a)	2	3.3	3.5	2.8	2	1.7	0.8	5.3	5.2	3.6	0.245	0.346	0.556	0.382
Exposed +0 % naltrexone (fig 3 a)	3	4.1	3.6	4.1	1.1	0.7	1.4	5.2	4.3	5.5	0.577	0.674	0.491	0.581
Exposed +food + 0.05% naltrexone (fig 3 b)	1	2.1	2.8	1.9	2.6	3	2.4	4.7	5.8	4.3	-0.106	-0.034	-0.116	-0.086
Exposed +food + 0.05% naltrexone (fig 3 b)	2	2.7	2.2	2.7	2.7	3	2.8	5.4	5.2	5.5	0.000	-0.154	-0.018	-0.057
Exposed +food + 0.05% naltrexone (fig 3 b)	3	2.5	3.1	2.9	2.6	3.3	2.9	5.1	6.4	5.8	-0.020	-0.031	0.000	-0.017
Exposed + 0.05% naltrexone +food (fig 3 c)	1	3.8	2.8	3.6	1.2	1.2	1.4	5.0	4.0	5.0	0.520	0.400	0.440	0.453
Exposed + 0.05% naltrexone +food (fig 3 c)	2	4.2	3.2	3.4	1.1	1.3	0.8	5.3	4.5	4.2	0.585	0.422	0.619	0.542
Exposed + 0.05% naltrexone +food (fig 3 c)	3	4.1	3.1	3.8	1.3	1.1	1	5.4	4.2	4.8	0.519	0.476	0.583	0.526

Each of the data points represents the volume in ul consumed by the flies from the capillaries containing the same type of food after substraction of food evaporation estimated in a separate tube with no flies. Each tube had two capillaries containing food only, two capillaries containing food plus alcohol (15%) and 6-8 flies. For each condition, three independent repeats each with triplicate tubes were carried out.
